# Beneficial Effect of a Fermented Wheat Germ Extract in Intestinal Epithelial Cells in case of Lipopolysaccharide-Evoked Inflammation

**DOI:** 10.1155/2020/1482482

**Published:** 2020-08-05

**Authors:** Z. Karancsi, A. V. Móritz, N. Lewin, A. M. Veres, Á. Jerzsele, O. Farkas

**Affiliations:** Pharmacology and Toxicology Department, University of Veterinary Medicine, Budapest, Hungary

## Abstract

In this study, the protective effect of a fermented wheat germ extract (FWGE) against LPS-induced inflammation and oxidative stress in IPEC-J2 porcine intestinal epithelial cells was studied. Enterocytes were treated with LPS derived from *Salmonella enterica* ser. Typhimurium and *Escherichia coli* O55:B5, O111:B4, and O127:B8 strains. Intracellular ROS level and extracellular H_2_O_2_ level were followed up by two fluorescent assays (DCFH-DA and Amplex Red). The effect of FWGE on the intestinal barrier integrity was determined by transepithelial electric resistance measurements and using a FD4 fluorescent tracer dye. IL-6 concentration of supernatants was also measured by the ELISA method. Our data revealed that FWGE had a significant lowering effect on the inflammatory response especially related to oxidative stress. Treatment with FWGE (1-2%) significantly decreased the level of intracellular ROS compared to LPS-treated cells. Furthermore, LPS-triggered partial disruption of epithelial integrity was reduced after FWGE application.

## 1. Introduction

The intestinal epithelium serves as a primary physical barrier against invading bacteria, toxins, and various environmental pollutants; however, it also plays an active role in the maintenance of absorption, regulation of barrier function, and immune homeostasis [1]. Gut-derived infections may be triggered by lipopolysaccharide (LPS) release from Gram-negative bacteria such as *Salmonella* spp. and *E. coli*. LPS can induce inflammatory responses, predominantly mediated by the activation of the NF-*κ*B pathway, contributing to proinflammatory cytokine (e.g., IL-6, IL-8, and TNF-*α*) overproduction, and it triggers the production of reactive oxygen species as well [2]. Infections caused by pathogenic bacteria and consequent inflammatory responses can cause human disease and can result in significant economic losses in animal industry.

In human healthcare, it is essential to promote prudent antibiotic use by preventing nosocomial infections and reduce overprescribing of antimicrobials. Most of the classes of antibiotics used for the treatment of bacterial infections in humans are also used in animals [3]. Misuse and overuse of antimicrobials in the treatment of human diseases and in animal husbandry can lead to bacterial resistance against clinically relevant drugs [4]. Therefore, the use of natural ingredients to improve gut health is of high importance. Gut health and intestinal barrier integrity could be improved and maintained with proper selection of feed additives which prevent pathogen invasion and inflammation, furthermore stimulating growth of useful microorganisms.

Food and feed supplements derived from plants often demonstrate various beneficial effects—they could have significant anti-inflammatory and antibacterial activity. A fermented wheat germ extract (FWGE) is a natural nontoxic substance which is already used in the human medicine as a supportive therapy in cancer patients under radiotherapy and chemotherapy [5]. The two major components to which beneficial effects are related, namely, 2-methoxy-benzoquinone and 2,6-dimethoxy-benzoquinone [6], are found in high concentrations in these extracts. FWGE has immunomodulatory [7], antiproliferative, antimetastatic, and antiangiogenic effects, and also, it can induce apoptosis in certain tumour cells like breast, colon, lung, and prostate cancer cells [8]. Lee et al. found that FWGE can promote apoptosis in different types of cancer cell lines such as SNU-5, MKN-45, SNU-620, SNU-1, and SNU-16. FWGE has significant antiproliferative effects and kills tumour cells by the induction of apoptosis via the caspase-poly (ADP-ribose) polymerase pathway [9]. Combined treatment of FWGE and 5-fluorouracil or dacarbazine resulted in a synergistic effect in the HCR-25 human cancer cell line and B16 murine melanoma cell line [6]. The effect of Avemar, which is a commercial dietary FWGE supplement for cancer patients, was also studied. Apoptosis induction in many Jurkat T leukaemia tumour cells [10], HL-60 human promyelocytic leukaemia cells [11], and tumour B and T cells [12] was also observed. FWGE has been also studied in autoimmune diseases. The anti-inflammatory effect of the FWGE extract was examined in rat adjuvant arthritis. It has been found that the FWGE treatment regulated inflammation-induced COX-1 and COX-2 expression and showed an additive effect with diclofenac [13]. Avemar can mitigate the clinical signs of systemic lupus erythematosus in mice via inhibiting Th2 response [14]. Although several studies have been performed on tumour and immune cells, the effect of this natural extract in the healthy gastrointestinal system is poorly examined.

IPEC-J2 cells are isolated from the jejunum of neonatal nonsuckled piglets [15]. These intestinal columnar porcine epithelial cells are noncancerous; therefore, they mimic the human physiological circumstances more closely compared to other transformed or tumorigenic cell lines. Using IPEC-J2 cells is a great tool to investigate epithelial integration, status of the antioxidant defense system, inflammation, and effects of pro- and prebiotics and other nutrients [16–18]. These cells can express and produce different types of inflammatory proteins (e.g., IL-6 and IL-8), cytokines (GM-CSF, TNF-*α*), receptors (toll-like receptors, F4 fimbrial receptors), and mucins [15]. Because swine and human intestinal functions are closely related, investigations performed on IPEC-J2 can provide reliable information regarding the pathogenesis of human intestinal infections [19].

In this study, our aim was to examine the anti-inflammatory and antioxidant effect of FWGE in jejunal epithelial cells under lipopolysaccharide stimulation. Moreover, the impact on the intestinal paracellular permeability of FWGE was also observed. To our knowledge, this is the first study, where an effect on reactive oxygen species and influence on the intestinal epithelial barrier of FWGE are studied on healthy intestinal epithelial cells.

## 2. Materials and Methods

### 2.1. Chemicals

FWGE was manufactured by Biropharma (Kunfehértó, Hungary) under the trade name *Immunovet*, and lipopolysaccharides (LPS) (derived from *Salmonella enterica* ser. Typhimurium, *Escherichia coli* O55:B5, *E. coli* O111:B4, and *E. coli* O127:B8, suitable for cell culture) were purchased from Sigma-Aldrich–Merck (Darmstadt, Germany).

### 2.2. Cell Line and Culture Conditions

The IPEC-J2 cell line was derived from the jejunum of a healthy neonatal piglet [20]. The cell culture was grown in the 1 : 1 mixture of Dulbecco's Modified Eagle's Medium and Ham's F-12 Nutrient Mixture (DMEM/F12) (plain medium) augmented with 5% foetal bovine serum (FBS), 5 *μ*g/ml insulin, 5 *μ*g/ml transferrin, 5 ng/ml selenium (ITS), 5 ng/ml EGF, and 1% penicillin-streptomycin (Sigma-Aldrich–Merck). IPEC-J2 cells were grown at 37°C in a humidified atmosphere of 5% CO_2_.

For the experiments, IPEC-J2 cells between passages 48 and 52 were seeded onto six-well polystyrene cell culture plates (Corning Inc., Corning, NY, USA), at a density of 1.5 × 10^5^ cells/ml (the volume of medium was 2 ml in each well according to the manufacturer's prescription). Cells were fed every second day until confluence was achieved.

### 2.3. Cell Viability Measurement by the Neutral Red Uptake Assay

Influence of FWGE on the viability of IPEC-J2 cells at different concentrations (1%, 2%, and 4%) and different durations of treatment (1 h, 2 h, and 24 h) was tested. The viability of the cells was also investigated at different concentrations (1, 10, and 20 *μ*g/ml) of the *Salmonella* LPS strains. FWGE was dissolved in distilled water, filtered with a 0.22 *μ*m membrane filter (Sigma-Aldrich–Merck, Darmstadt, Germany), and diluted in cell culture medium. LPS were freshly dissolved in plain medium. IPEC-J2 cells were seeded in a 96-well plate and incubated with FWGE for 1, 2, and 24 h, respectively. LPS strains were tested for a 1 h treatment period. The percentage of living cells was determined after 24 h of treatment by neutral red assay following the method of Repetto et al. [21].

### 2.4. Incubation of Enterocytes with LPS and FWGE

After IPEC-J2 monolayers have reached confluency, they were washed twice with plain medium. LPS derived from different bacterial strains (*Salmonella enterica* ser. Typhimurium, *Escherichia coli* O55:B5, *E. coli* O111:B4, and *E. coli* O127:B8) was used to evoke oxidative stress and inflammation. Control samples were treated with DMEM/F12 plain medium. LPS solutions were added to the plain medium at 10 *μ*g/ml concentration [17]. FWGE solutions were dissolved in distilled water and filtered with a 0.22 *μ*m membrane filter; thereafter, the solutions were diluted in the plain medium in two different concentrations (1% and 2%). After 1 h incubation with LPS, FWGE test compounds, and their combinations, cells were rinsed with plain medium and cultured for additional 24 h for redox status and inflammation studies.

### 2.5. Measurement of Intracellular ROS and Extracellular H_2_O_2_ Levels in IPEC-J2 Cells

IPEC-J2 cells were treated with the four different types of LPS (10 *μ*g/ml), the FWGE (1%, 2%), and their combinations in phenol red-free medium on twenty-four-well culture plates. Extracellular measurement of H_2_O_2_ was performed using the Amplex Red Hydrogen Peroxide/Peroxidase Assay Kit (Thermo Fisher Scientific, Waltham, USA) following the manufacturer's instruction [22]. Fluorescence intensity was measured at an excitation wavelength of 560 nm and an emission wavelength at 590 nm (Victor X2 2030 fluorometer, PerkinElmer, Waltham, MA, USA).

Change in the intracellular redox state of enterocytes was determined using a 2′,7′-dichloro-dihydro-fluorescein diacetate (DCFH-DA) dye (Sigma-Aldrich–Merck, Darmstadt, Germany). Intracellular ROS oxidize nonfluorescent DCFH-DA to the highly fluorescent dichlorofluorescein form (DCF) [23]. IPEC-J2 cells were treated with the LPS of different bacterial strains (10 *μ*g/ml) and with the FWGE (1%, 2%) in phenol red-free DMEM/F12 for 1 h. DCFH-DA (10 *μ*M) was added to IPEC-J2 cells for 30 minutes. Cells were rinsed with medium, scraped, and centrifuged for 10 minutes at 4500 rpm at 4°C. Fluorescence was determined with a Victor X2 2030 fluorometer at an excitation wavelength of 480 nm and an emission wavelength of 530 nm.

### 2.6. IL-6 ELISA

IPEC-J2 cells were treated with the four different types of LPS (10 *μ*g/ml), the FWGE (1%, 2%), and their combinations in phenol red-free medium on twenty-four-well culture plates for 1 h. After 1 h treatment, solutions were removed and cell culture medium was added to the IPEC-J2 cell. Supernatants were collected after 6 h, and IL-6 concentrations were measured from 100 *μ*l of samples. Each sample was measured twice. The level of IL-6 secretion (pg/ml) was measured with porcine-specific IL-6 ELISA kits (Sigma-Aldrich–Merck, Darmstadt, Germany) following the manufacturer's guide.

### 2.7. Paracellular Permeability Measurement

IPEC-J2 cells were seeded on 6-well polyester membrane inserts and were grown to confluent, differentiated monolayers. LPS derived from *S.* Typhimurium was added at 10 *μ*g/ml concentration for 1 h to cells, and transepithelial electric resistance (TEER) values were measured prior to and 2, 4, and 24 h after LPS treatment. Simultaneously with LPS administration, cells were treated with 1 mg/ml fluorescein isothiocyanate-dextran 4 kDa (FD4) tracer dye (Sigma-Aldrich, Darmstadt, Germany) with different incubation times (2, 4, and 24 h). Medium samples from the basolateral chambers were collected, and the FD4 concentration was determined by a fluorescent method at excitation 485 nm and emission 535 nm (PerkinElmer, Victor X2 2030 fluorimeter).

### 2.8. Statistics

Statistical analysis of our data was performed with R 3.3.2 (2016) software (R Foundation, Vienna, Austria). Differences between means were determined by two-way ANOVA, with data of normal distribution, and homogeneity of variances was also confirmed. To analyse treated groups to controls, the Dunnett post hoc test was applied and the Fisher LSD test was used to compare different treatments. Differences between treatment groups were considered proven if *p* values were <0.05.

## 3. Results

### 3.1. Viability of IPEC-J2 Cells

**Figure 1:**
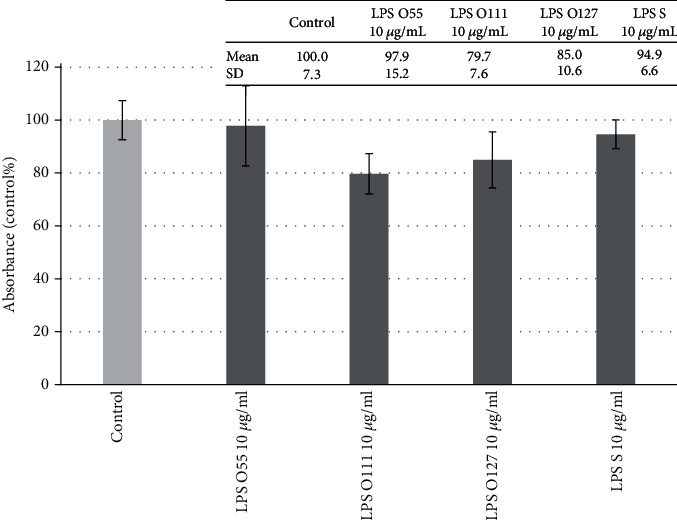
Viability of porcine intestinal epithelial cells (IPEC-J2) after treatment of different types of lipopolysaccharides (LPS) from *Salmonella* Typhimurium (LPS S), *E. coli* O55:B5 (LPS O55), *E. coli* O111:B4 (LPS O111), and *E. coli* O127:B8 (LPS O127) in 10 *μ*g/ml concentration. Data are shown as means with standard deviations (*n* = 6/group) in control percentage. There was no significant alteration in cell viability at 95% confidence level (*p* < 0.05).

To select the proper concentration of LPS for the experiments without reduction of cell viability, the neutral red uptake assay was performed. There was no significant reduction regarding cell viability after treatment with the highest LPS concentration (20 *μ*g/ml) of *E. coli* O55:B5, O111:B4, and O127:B8 (data not shown). LPS from *S.* Typhimurium did not cause any significant alteration in cell viability at concentration of 10 *μ*g/ml which was also true in the case of different *E. coli* strains (Figure 1). Based on the abovementioned results, 10 *μ*g/ml LPS concentration was chosen for further experiments.

**Figure 2:**
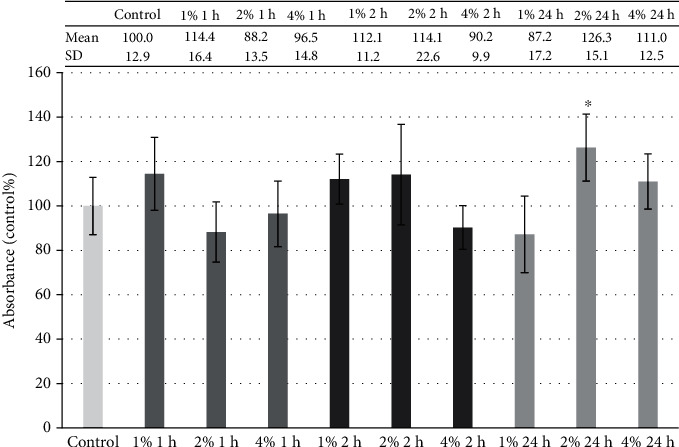
Viability of porcine intestinal epithelial cells (IPEC-J2) after treatment at different concentrations (1%, 2%, and 4%) of FWGE (duration of 1 h, 2 h, and 24 h). Data are shown as means with standard deviations (*n* = 6/group; ^∗^*p* < 0.05) in control percentage.

FWGE showed no significant reduction in cell viability (Figure 2); moreover, in the case of the 2% FWGE 24 h treatment, the number of living IPEC-J2 cells was higher compared to that of the control. For further experiments, 1% and 2% FWGE was used for 1 h.

### 3.2. ROS Production in IPEC-J2 Cells after Treatment with FWGE

**Figure 3:**
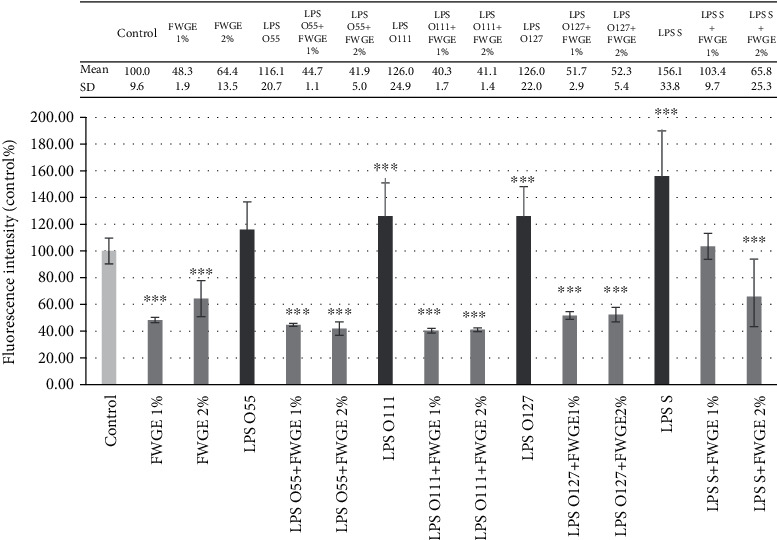
Intracellular reactive oxygen species level in porcine intestinal epithelial cells (IPEC-J2) after treatment with different types of lipopolysaccharides (LPS) from *Salmonella* Typhimurium (LPS S), *E. coli* O55:B5 (LPS O55), *E. coli* O111:B4 (LPS O111), and *E. coli* O127:B8 (LPS O127) in 10 *μ*g/ml concentration; FWGE (FWGE) in 1% and 2% concentrations; and their combinations. Data are shown as means with standard deviations (*n* = 6/group; ^∗∗∗^*p* < 0.001) in control percentage and are compared to those of the control group.

Stimulation of the IPEC-J2 cells with different LPS types caused significant increase in intracellular ROS level compared to the control group except for *E. coli* O55:B5 LPS treatment (Figure 3). FWGE *per se* in both applied concentrations (1% and 2%) significantly decreased the intracellular quantity of reactive oxygen species compared to the control group. Significant decrease in intracellular ROS level was also observed after the combined treatment of LPS from all *E. coli* types and FWGE. Only 1% FWGE did not cause significant reduction in the elevated ROS level after *S.* Typhimurium LPS treatment.

**Figure 4:**
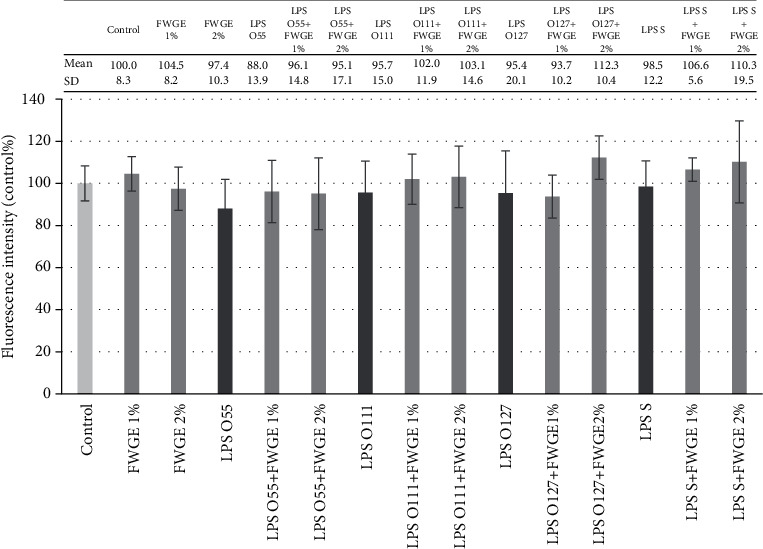
Extracellular hydrogen peroxide (H_2_O_2_) level in porcine intestinal epithelial cells (IPEC-J2) after treatment with different types of lipopolysaccharides (LPS) from *Salmonella* Typhimurium (LPS S), *E. coli* O55:B5 (LPS O55), *E. coli* O111:B4 (LPS O111), and *E. coli* O127:B8 (LPS O127) in 10 *μ*g/ml concentration; FWGE (FWGE) in 1% and 2% concentrations; and their combinations. Data are shown as means with standard deviations (*n* = 6/group; ^∗^*p* < 0.05) in control percentage and are compared to those of the control group.

Extracellular H_2_O_2_ levels are shown in Figure 4. Treatment with different LPS types did not indicate significant increase in H_2_O_2_ compared to the control group. FWGE with combinations of different types of LPS could not influence the extracellular hydrogen peroxide quantity in the samples.

### 3.3. IL-6 Production in IPEC-J2 Cells after Treatment with FWGE

**Figure 5:**
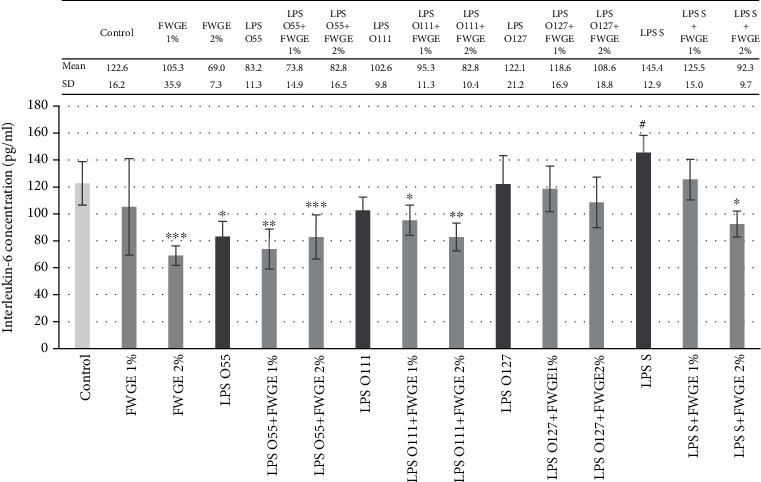
Interleukin-6 (IL-6) concentration in porcine intestinal epithelial cells (IPEC-J2) after treatment with different types of lipopolysaccharides (LPS) from *Salmonella* Typhimurium (LPS S), *E. coli* O55:B5 (LPS O55), *E. coli* O111:B4 (LPS O111), and *E. coli* O127:B8 (LPS O127) in 10 *μ*g/ml concentration; FWGE (FWGE) in 1% and 2% concentrations; and their combinations. Data are shown as means with standard deviation (*n* = 6/group; ^#^*p* < 0.1; ^∗^*p* < 0.05; ^∗∗^*p* < 0.01; ^∗∗∗^*p* < 0.001) and are compared to those of the control group.

The LPS types derived from different *E. coli* strains did not provoke a significant elevation of IL-6 concentration in IPEC-J2 cells (Figure 5). LPS derived from *S.* Typhimurium slightly increased the IL-6 concentration (*p* = 0.09) compared to the control group. The combination of LPS of *S.* Typhimurium and *E. coli* O111:B4 origin with FWGE significantly decreased the IL-6 concentration. Interestingly, LPS from *E. coli* O55:B5 caused significant decrease in IL-6 concentration *per se*, as well as its combinations with FWGE. LPS of *E. coli* O127:B8 origin did not influence the IL-6 concentration.

### 3.4. Paracellular Permeability of IPEC-J2 Cells after LPS and FWGE Treatment

**Figure 6:**
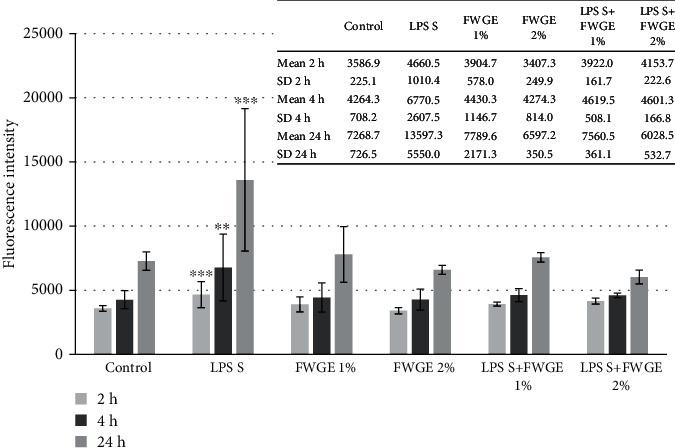
Penetration of FD4 from the apical to the basolateral compartment of IPEC-J2 cells after LPS treatment (10 *μ*g/ml, treatment time 1 h, detection after 2 h, 4 h, and 24 h). Effect of FWGE on the paracellular permeability. Data are shown as means with standard deviations (*n* = 3/group; ^∗∗^*p* < 0.01; ^∗∗∗^*p* < 0.001) and are compared to those of the control group.

After 2, 4, and 24 h LPS treatment, partial disruption of the epithelial cell layer was observed. FD4 fluorescence intensity in the basolateral chamber was significantly increased in the LPS-treated samples compared to the untreated samples (Figure 6). The fluorescence intensity was not altered significantly after treatment with FWGE (1% and 2%) only. After simultaneous treatment of IPEC-J2 cells with LPS (10 *μ*g/ml) and FWGE in different concentrations (1% and 2%), the presence of the FD4 tracer in the basolateral compartment significantly decreased. TEER values were also measured before and after LPS treatment to check the integrity of the polarized IPEC-J2 monolayer. It was observed that neither LPS nor FWGE affects TEER values significantly (data not shown).

## 4. Discussion

In recent years, avoiding unnecessary use of antibiotics in human and veterinary medicine is becoming increasingly important. Low-dose dietary antibiotics, in the context of growth promotion in livestock, have been banned because of cross resistance against human critically important antimicrobials. Considering the abovementioned facts, there is a growing interest to replace antibiotics in the veterinary practice with certain natural alternatives. Fermented plant extracts are common subjects of these studies because of their many positive effects on bacteria-induced inflammation and intestinal barrier impairment. Among others, Bose and Kim demonstrated that fermented preparation of *Rhizoma atractylodis macrocephalae* reduced COX-2, IL-1*β* and IL-6, and TNF-*α* expression in *P. aeruginosa* LPS-treated macrophages [24].

FWGE is becoming a frequently applied dietary supplement in human cancer patients. Application of FWGE is increasing not only in the human but also in the veterinary medicine in order to improve the animal immune system. It is supposed that its active ingredients have anti-inflammatory and antioxidant activity. In lipopolysaccharide-induced mouse macrophages, treatment with FWGE in combination with citric acid resulted in decreased secretion of inflammatory cytokines TNF-*α*, IL-6, and IL-12 and reduced synthesis of COX-2 via the inhibition of phosphorylation of NF-*κ*B and p38, while FWGE *per se* decreased IL-12 production only. Both treatment types increased the level of the anti-inflammatory cytokine IL-10 and heme oxygenase-1 significantly [25]. Phagocytic activity and phagocytic index were significantly enhanced in the case of growing pigs fed with FWGE [26]. The effect of fermented wheat germ was tested in an experimental rat model of cardiovascular remodelling. Treatment with Avemar improved cardiac function and attenuated the increased plasma level of the oxidative stress marker malondialdehyde [27].

Nevertheless, there is not enough data available about the anti-inflammatory effect of this product, and the mechanisms behind the positive effects are not known. Moreover, there is not enough available information about the efficacy of FWGE on a healthy intestinal epithelium. The intestinal wall is the first protecting layer against the microbes or harmful toxic substances. The protection is performed by the physical barrier which contains epithelial cells and the tight junction connection between them, the immune cells, and the mucin layer [28]. In our examination, we modelled oxidative stress in healthy porcine epithelial cells challenged with lipopolysaccharides derived from different Gram-negative bacteria.

Our study showed that FWGE in different concentrations (1%, 2%, and 4%) resulted in no cell death; moreover, in 2% concentration after 24 h treatment, improved cell viability can be observed. Concentration-dependent increase in viability in IPEC-J2 cells can be observed after treatment with natural plant compounds such as *Hippophae rhamnoides* polysaccharides [29] or rosmarinic acid [30].

It has been proven that under *in vitro* conditions, FWGE has an antioxidant effect in porcine epithelial cells when oxidative stress is triggered by LPS from *S.* Typhimurium and different *E. coli* (O55:B5, O111:B4, and O127:B8) strains. Intracellular ROS level was reduced significantly by the FWGE; therefore, this dietary supplement can protect the intestinal cells from oxidative stress caused by a Gram-negative bacterial wall component. There was no difference in the antioxidant activity of different FWGE solutions in the case of *E. coli* LPS-provoked inflammation; however, in the case of *S.* Typhimurium LPS treatment, we found that 2% FWGE reduced intracellular ROS level more effectively than 1% FWGE treatment. Extracellular hydrogen peroxide concentration did not show evaluable alteration neither with FWGE nor with different types of LPS treatment.

It is known that not only the immune cells but also the intestinal epithelial cells play a crucial role in the mucosal immune response [31]. We found that LPS from *S.* Typhimurium could slightly induce IL-6 secretion in IPEC-J2 cells. In the presence of 2% FWGE (combined treatment with LPS *S.* Typhimurium), reduced IL-6 production can be observed (*p* < 0.001). *E. coli* LPS did not cause a significant elevation in IL-6 concentration in porcine epithelial cells. The explanation of this phenomenon requires further experiments, but unresponsiveness of the IPEC-J2 cell line for some stimuli can be sometimes observed. For example, after *S.* Typhimurium LPS treatment, the mRNA level of TLR-4 was not changed [16]. This result refers to those found in the case of some human epithelial cell lines, which show a low-level expression of TLR-4. This phenomenon was explained by their relative resistance to the permanent exposure to Gram-negative commensal bacteria [32].

Tight junctions are multiprotein complexes between adjacent epithelial cells; they control the paracellular transport and maintain cell polarity. Altered tight junction structure can result in the disruption of the intestinal barrier and loss of cell polarity and leads to facilitated translocation of bacteria and bacterial products from the lumen to the gut [33]. In this study, LPS treatment resulted in an increased quantity of the FD4 fluorescent dye in the basolateral compartment without influencing TEER values of the IPEC-J2 cell layer. The same fact was observed in our previous research [34]. It can be concluded that two paracellular pathways exist: an ionic charge-selective small-pore system carrying most of the electrical current (reflected in TEER) and larger barrier discontinuities lacking charge and size discrimination in epithelia. Disruption of the epithelial layer caused by *S.* Typhimurium LPS treatment was stopped by the application of FWGE treatment in the IPEC-J2 cells.

## 5. Conclusions

We described first that FWGE has many beneficial activities in the intestinal cells in case of LPS-evoked oxidative damage. FWGE did not cause cell death in different concentrations (1%, 2%, and 4%), and it can decrease intracellular ROS level after LPS-induced oxidative stress. In addition, it has a protective effect on cell layer integrity in the case of *S.* Typhimurium LPS treatment. Our results suggest that FWGE could be a promising compound in the prevention and treatment of intestinal impairment caused by bacteria in both human and veterinary medicine.

## Data Availability

All the data used to support the findings of this study are available from the corresponding author upon request.
